# Impact of Proton Irradiation Depending on Breast Cancer Subtype in Patient-Derived Cell Lines

**DOI:** 10.3390/ijms251910494

**Published:** 2024-09-29

**Authors:** Marika Musielak, Kinga Graczyk, Małgorzata Liszka, Athanasia Christou, Monika A. Rosochowicz, Michał S. Lach, Beata Adamczyk, Wiktoria M. Suchorska, Tomasz Piotrowski, Bo Stenerlöw, Julian Malicki

**Affiliations:** 1 Department of Electroradiology, Poznan University of Medical Sciences, 61-701 Poznan, Poland; wiktoria.suchorska@wco.pl (W.M.S.); tomasz.piotrowski@wco.pl (T.P.); julian.malicki@wco.pl (J.M.); 2 Doctoral School, Poznan University of Medical Sciences, 61-701 Poznan, Poland; monika.rosochowicz@wco.pl; 3 Radiobiology Laboratory, Department of Medical Physics, Greater Poland Cancer Centre, 61-866 Poznan, Poland; michal.lach@wco.pl; 4 Clinical Dosimetry, Department of Medical Physics, Greater Poland Cancer Centre, 61-866 Poznan, Poland; kinga.graczyk@wco.pl; 5 The Skandion Clinic, 751 23 Uppsala, Sweden; malgorzata.liszka@skandion.se (M.L.); athanasia.christou@skandion.se (A.C.); 6 Department of Orthopaedics and Traumatology, Poznan University of Medical Sciences, 61-701 Poznan, Poland; 7 Breast Surgical Oncology Department, Greater Poland Cancer Centre, 61-866 Poznan, Poland; beata.adamczyk@wco.pl; 8 Department of Medical Physics, Greater Poland Cancer Centre, 61-866 Poznan, Poland; 9 Department of Immunology, Genetics and Pathology, Rudbeck Laboratory, Uppsala University, 753 10 Uppsala, Sweden; bo.stenerlow@igp.uu.se

**Keywords:** proton therapy, breast cancer, oncology, radiobiology, tumor microenvironment

## Abstract

**Simple Summary:**

Patient-specific factors must be thoroughly analyzed to minimize the side effects of applied treatments. This study examined the radiobiological response of established and patient-derived malignant cell lines, cancer-associated fibroblasts, and skin fibroblasts to proton irradiation (IRR). Using clonogenic assay, γH2AX, and p53 staining, it was found that breast cancer cell lines of different subtypes had similar responses. However, cancer-associated fibroblasts from TNBC tumors were more resistant than those from luminal A tumors. Skin fibroblasts responded uniformly across all doses. The responses of patient-derived cell lines suggested that each patient may have a distinct radiotherapy result due to specific tumor microenvironmental characteristics.

**Abstract:**

Research on different types of ionizing radiation’s effects has been ongoing for years, revealing its efficacy in damaging cancer cells. Solid tumors comprise diverse cell types, each being able to respond differently to radiation. This study evaluated the radiobiological response of established (MDA-MB-231 (Triple negative breast cancer, TNBC), MCF-7 (Luminal A)) and patient-derived malignant cell lines, cancer-associated fibroblasts, and skin fibroblasts following proton IRR. All cell line types were irradiated with the proton dose of 2, 4, and 6 Gy. The radiobiological response was assessed using clonogenic assay, γH2AX, and p53 staining. It was noticeable that breast cancer lines of different molecular subtypes displayed no significant variations in their response to proton IRR. In terms of cancer-associated fibroblasts extracted from the tumor tissue, the line derived from a TNBC subtype tumor demonstrated higher resistance to ionizing radiation compared to lines isolated from luminal A tumors. Fibroblasts extracted from patients’ skin responded identically to all doses of proton radiation. This study emphasizes that tumor response is not exclusively determined by the elimination of breast cancer cells, but also takes into account tumor microenvironmental variables and skin reactions.

## 1. Introduction

Radiotherapy (RT) plays an important role in the treatment of breast cancer (BC) [1]. Increasingly, proton RT is being considered as a valuable option for treating challenging tumors due to its favorable dose-depth distribution [2]. It is especially pertinent in BC patients where the target is near the heart and lungs [3]. Proton beams enable precise delivery of high energy doses to the tumor while sparing vital organs, crucially reducing cardiotoxicity [4]. Apart from considering anatomical nuances, it is important to underscore the significance of molecular factors influencing the tumor’s radiobiological response. Biological aspects such as various molecular subtypes of BC and the tumor microenvironment (TME) originating from specific tumors may also be different and may influence the response after RT. Therefore, it is necessary to analyze such aspects in the context of patient-specific factors. Analyzing individual patient characteristics should guide the improvement of treatment results and allow for RT personalization. However, there is a notable knowledge gap considering the importance of evaluating BC response through patient-specific cancer models that include molecular factors from the surrounding environment.

Numerous studies have illuminated the intricate relationship between cancer and the cells comprising the TME [5,6]. Among these, cancer-associated fibroblasts (CAFs) represent the predominant group of cells creating tumor surroundings [7]. Distinguished morphologically and physiologically from their dermal counterparts, CAFs possess secretory capabilities that drive tumor progression, fostering their growth and propensity for metastasis [8]. Furthermore, they engage in intricate communication with other TME constituents, including endothelial and immune cells [9]. While the radiobiological response of the BC after proton irradiation is often focused on the tumor cells alone, its impact on the broader TME remains elusive. A detailed examination of CAF responses could be crucial in understanding BC proton treatment efficacy.

Depending on the presence or absence of various receptor expressions on the cancer cell surface, different molecular subtypes of BC are distinguished. It has been demonstrated that some primary subtypes can be reliably determined by solely utilizing genes associated with estrogen receptor (ER), progesterone receptor (PR), and human epidermal growth factor receptor 2 (HER-2) characteristics [10]. These subtypes are categorized as ER-negative/PR-negative/HER2-negative (basal-like, triple-negative breast cancer (TNBC)), HER2-positive (HER2-enriched), and ER-positive/HER2-negative (luminal A and B).

Personalized biological models for assessing the post-IRR response are garnering escalating interest [11]. The latest scientific articles stress the importance of considering individual patient-specific factors [12,13]. Patient-derived cell lines prove invaluable in predicting treatment response [14]. Conventional models involve cell lines isolated from cancer tissue, which were genetically modified to establish a standardized platform for scientific comparison. Due to its limitations, there is a growing emphasis on utilizing patient tissue-derived lines as the most reliable predictor of treatment response [15]. On the other hand, such models present tremendous challenges associated with the difficulty and expense of isolation, as well as preserving the original characteristics of the primary cell lines [16].

This study aimed to evaluate the radiobiological response of established BC cell lines of two different molecular subtypes, luminal A and TNBC; cell lines isolated from patients’ tumor tissue: cancerous cell line and CAF cell lines; and normal fibroblast (NF) cell lines isolated from patients’ skin. An important aspect of the assessment was the analysis of the radiobiological response of BC molecular subtypes after proton beam radiation.

## 2. Results

### 2.1. Obtaining a Biological Model Consisting of Primary Cell Cultures

**Figure 1:**
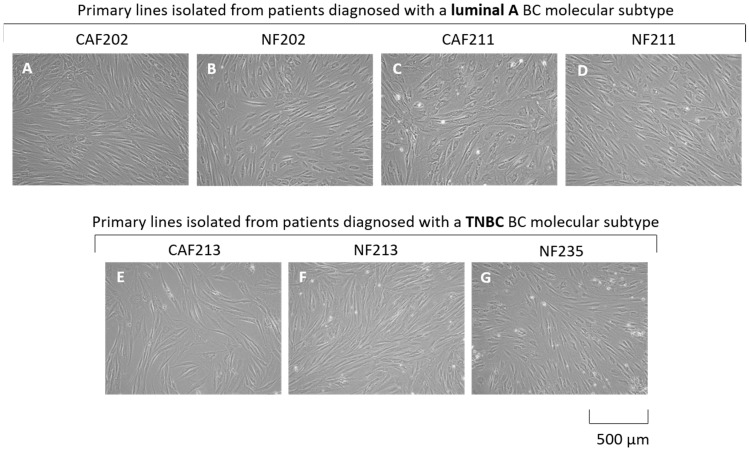
Phase-contrast images determine the morphology of isolated cell lines ((**A**) CAF 202, (**B**) NF202, (**C**) CAF211, (**D**) NF211, (**E**) CAF213, (**F**) NF213, (**G**) NF235). They represent an elongated, spindle-like shape morphology corresponding to a fibroblast-like phenotype. Pictures were taken at 100× magnification.

**Figure 2:**
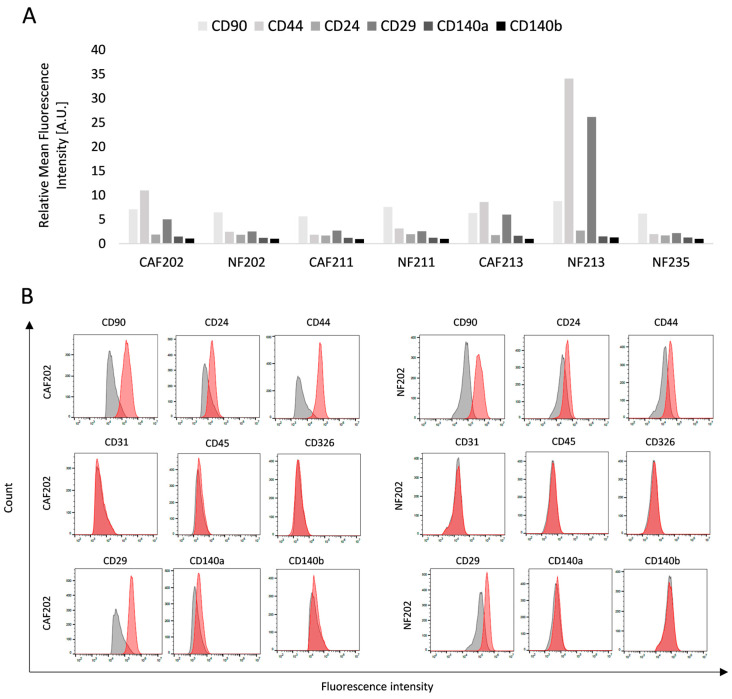
Flow cytometric analysis of the phenotype of isolated CAF and NF lines. The biomarker expression indicates specific cell line characteristics, confirming its phenotype and molecular subtype. For cell phenotyping, CD90, CD44, CD24, CD29, CD140a, and CD140b biomarkers were used. (**A**) Plot of each cell line’s relative mean fluorescence intensity of selected biomarkers. (**B**) Representative histograms of CAF and NF isolated from the same patient (no. 202) show the fluorescence intensity level. The gray histogram determines unstained cells (negative group), while the red one marks labeled cells with a specific antigen. The presented results are based on a single experiment.

**Figure 3:**
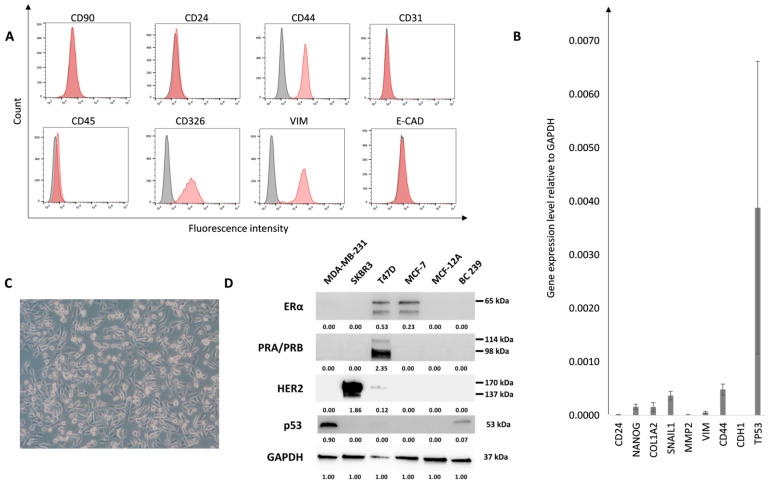
Characterization of the BC239 cell line. (**A**): The BC239 line confirmation of BC origin using flow cytometry. The lack of CD90, CD31, and CD45 expression demonstrated exclusion of fibroblast, leukocyte, and endothelial features. The presence of CD44, CD326, and VIM and the lack of E-CAD and CD24 show the increased mesenchymal-like population with a high content of CSC originating from the epithelium. Grey histograms represent isotype control and red histograms correspond to the staining antigen of interest. (**B**): The relative gene expressions of the BC239 line. The genes characteristic for BC cells and TME were chosen. (**C**): The microscopic evaluation of BC239 cells showing spindle-like morphology. The pictures were taken using an inverted microscope Axio Vert.A1 (Carl Zeiss, Germany). Magnification ×100. (**D**): The evaluation of receptor status and p53 expression of the isolated primary breast cancer cell line using the Western blot technique. The blots represent the status of ERα, PRA/PRB, HER2 receptors, and p53 in commercially available breast cancer cell lines (MDA-MB-231, SKBR3, T47D, MCF-7), normal epithelial breast cell line (MCF12A), and the primary breast cancer cell line (BC239). GAPDH was used as a loading control. Below the blots, the signal intensity values normalized to GAPDH are shown.

In this study, a new BC239 cell line was obtained to observe the proton RT outcome considering patient specificity and compare results with established commercially available BC cell lines. In addition, CAF lines were isolated to observe the radiobiological response of cells creating BC stroma, a crucial element of TME. Their healthy analog, NF lines, from the same patients’ skin were used to give information on how cancer cells could mitigate responsiveness to radiation as CAFs. The aforementioned cell lines were obtained based on our previous isolation optimization [16] and phenotyping method [12], and the results are described in detail below (Figure 1, Figure 2 and Figure 3). Firstly, NF and CAF cells’ morphology was determined under the microscope, and no significant differences were observed (Figure 1).

For further evaluation, all isolated NFs and CAFs cell lines were phenotyped using flow cytometry to confirm their primary origin and give insight into their differences, besides the primary lack of morphological overlook (Figure 2A). Isolated cells were positive for CD90, indicating their fibroblast origin, whereas they were negative for CD31, CD45, and CD326, demonstrating that they were not endothelium, leukocytes, or epithelium related, respectively. The CD24 and CD44 expressions were quite heterogeneous among isolated cell lines. The functional aspects of CD24 are achieved through binding with ligands or involvement in signal transduction processes, which facilitate the induction and advancement of tumors [17]. CD44 plays a role in the mechanisms of drug resistance in cancer cells. The research indicates that CD44 in CAFs is active in sustaining cancer stem cell populations within the tumor microenvironment [18]. There was no indication of any pattern according to those biomarkers. Other molecules such as CD29, CD140a, and CD140b were chosen as biomarkers determining the molecular subtypes of cells. CD29 is an anti-human integrin β1-subunit antibody [19] and was described as a CAF subgroup indicator [20]. Platelet-derived growth factor receptor alpha (CD140a) and beta (PDGFR) (CD140b) are considered typical indicators of interstitial resident fibroblasts [21]. There were no statistically significant differences between CAF and NF when analyzing the expression of those biomarkers. The noticeable higher values of CD44 and CD29 were observed in NF213 which probably suggests patient-specific features. The representative cytometric histograms (CAF and NF isolated from patient no. 202) of all biomarkers are presented in Figure 2B. Histograms from each phenotyped line are provided in the Supplement Materials.

One of the goals of this study was also to test the effect of proton IRR on established primary breast cancer cells. We were able to establish a cell line named BC239 from patient no. 239. The isolated cells proliferated quickly, and their phenotype remained intact for several passages. The molecular and macroscopic evaluation was performed to characterize this cell line (Figure 3A–D). After an isolation process, no population of CAF cells was observed during cell culture, which was confirmed by flow cytometry analysis and lack of CD90 presence (Figure 3A). Moreover, the high content of CD44 and lack of CD24 were confirmed, which is related to cancer-like stem cell (CSC) features. A lack of endothelial and leukocytes (CD31 and CD45 negative) was observed among these cells. The BC239 cells were positive for CD326 (EpCAM, a tumor-associated antigen, showing the epithelial origin [22]) and vimentin (VIM, a marker of EMT indicating possible metastatic/migratory properties) with a parallel lack of E-Cadherin. Additionally, based on our previous article [16], we confirmed the aforementioned flow cytometric analysis with the panel of genes characteristic of BC cells (Figure 3B). We observed an expression of CD24, CD44, and NANOG, indicating a highly potent capacity to rebuild the tumor mass. We have also confirmed a mesenchymal-like state by the presence of SNAIL, VIM, and MMP2 expression and a lack of E-Cadherin. In addition, we have observed medium expression of COL1A2, which is a marker of TME. Among that panel, we also tested Tp53, which reached the highest expression level. Its presence is related to the response to the treatment, especially for the DNA damage response signaling pathway. For the receptor status of the isolated BC239, Western blot analysis was performed (Figure 3D). We compared this cell line with commercially available cell lines representing different histological surrogates ERα, PRA/PRB, and HER2 as follows: MDA-MB-231—triple-negative, SKBR3—HER2 positive, T47D and MCF-7 Luminal A, MCF-12A—normal epithelial cell. Surprisingly, we could not confirm the Luminal A status, provided by clinicopathological status from the treated specimen’s medical history. Unfortunately, we could not isolate NF from a 239 patient’s breast skin. Despite this, and taking all the data all together, we have confirmed the malignant nature of the new established BC cell line, as well as its purity.

### 2.2. Survival Fraction (SF): Heterogenic Radiobiological Response

**Figure 4:**
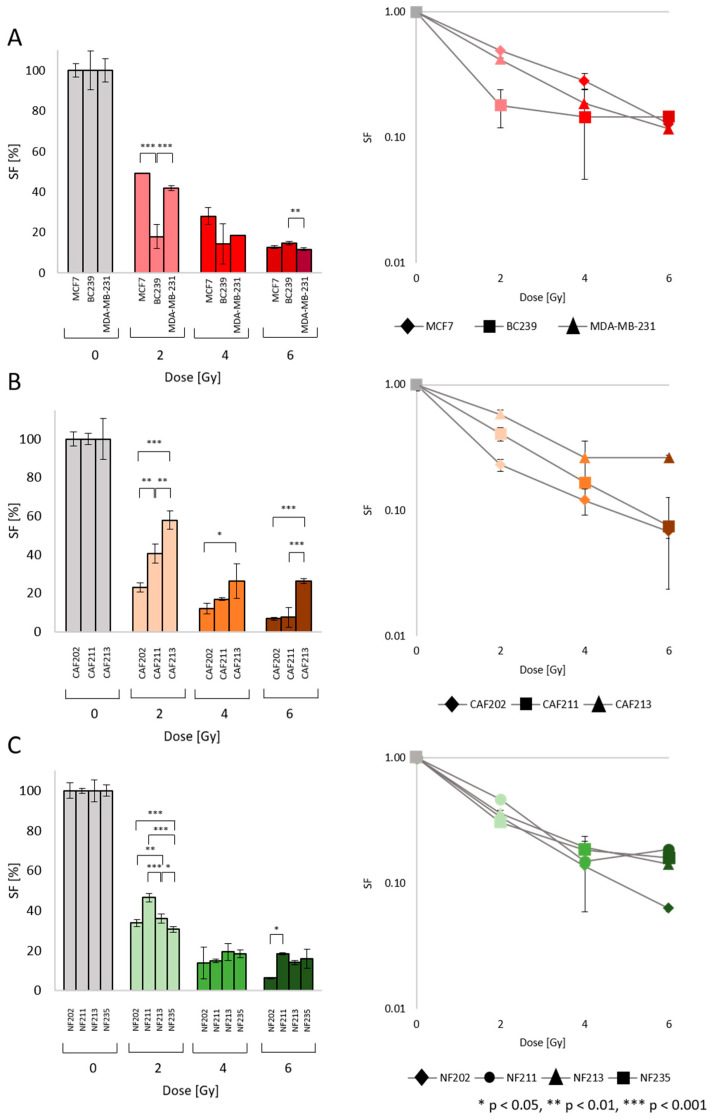
Cell survival fraction (SF) post proton IRR. All cell lines were irradiated with 2, 4, and 6 Gy doses. The column graphs (left side) show the statistically significant differences between the cell lines for each dose. The curve plots (right side) were determined to illustrate the exponential dependency between the SF and dose. The various cell lines were: BC (**A**) (red), CAF (**B**) (orange), and NF (**C**) (green). The *p*-value was adjusted to * *p* < 0.05, ** *p* < 0.01, and *** *p* < 0.001. The values presented in the graph are the mean of the obtained results and the deviation is the standard deviation. The experiment was performed in triplicate.

The clonogenic assay was carried out to determine the SF of irradiated cells (Figure 4A–C). All cell lines were irradiated with a proton beam using 2, 4, and 6 Gy doses. With increasing IRR dose, the SF of all tested cells decreased. However, the irradiated BC cell lines MCF7, MDA-MB-231 (established cell lines), and BC239 (primary cell line) indicated different responsiveness (Figure 4A). The BC239 cell line gained similar SF levels after IRR with each dose and the survival levels were 18% post 2 Gy and 15% post 4 Gy and 6 Gy, respectively. The established cell lines responded similarly but they were more resistant than the BC239 cells to 2 Gy. A statistically significant difference was observed between the BC239 line and MCF as well as MDA-MB-231, especially at 2 Gy (*p* = 0.0002, and *p* = 0.0005, respectively). At 4 Gy, there was no indication of differences, in contrast to at 6 Gy, where lower SF was obtained by MDA-MB-231 cells compared to the BC239 (*p* = 0.008). One of the possible explanations could be related to the p53 status of tested cells, showing that our primary cell line still has a functioning mechanism of DNA damage response (DDR) response.

As we suspected, the different tumor types will affect the responsiveness of the cells building stroma to IRR (Figure 4B). In the analysis, CAF202 and CAF211 were the representatives isolated from BC of the luminal A subtype, whereas CAF213 was isolated from TNBC. Analyzing SF post 2 Gy of IRR, the most radiation-sensitive CAF line was CAF202, whereas the most resistant was CAF213. Even if the CAF lines were isolated from tissues of the same BC molecular subtype, the diversity in results after 2 Gy was noticed, which could be expected since this is probably a patient-specific response. Moreover, statistically significant differences between CAF13 and CAF202 and CAF211 were observed (*p* = 0.002, *p* = 0.005). Interestingly, after higher IRR doses, there were no alterations between CAF202 and CAF211. The CAF213, as the most IRR-resistant one, indicated divergence compared to CAF202 (*p* = 0.045) after the dose of 4 Gy. However, post 6 Gy IRR CAFs isolated from the same molecular subtype of cancer demonstrated 7% of SF, whereas CAF213 showed 26%, determining the statistical differences (*p* = 0.001 for both CAFs isolated from luminal A tumors).

The NF cell lines were irradiated with the same IRR scheme to observe the response of skin fibroblasts. To show that the neighboring effect with malignant cells mostly caused the response to IRR in CAFs. They were isolated from the skin of 4 patients diagnosed with luminal A (2 patients) and TNBC (2 patients) tumors. Post 2 Gy, there was no difference between NF202 and NF211 isolated from patients with luminal A cancer, but there was a diversity in SF comparing different molecular subtypes. The SF level of NF213 was higher than the SF level of NF202 (*p* = 0.003), while simultaneously lower by 10% compared to NF211 (*p* = 0.001). The lowest SF after 2 Gy was observed in NF235 (31%). The statistical diversities between NF235 and NF202, NF211, and NF213 were determined to be *p* = 0.0001, *p* = 0.0001, and *p* = 0.032 respectively. Contrary to differences noticed after 2 Gy of IRR, there was no statistical diversity in SF level between NFs post 4 Gy. NFs isolated from patients diagnosed with luminal A tumors presented lower SF levels than NFs associated with TNBC. NF202 and NF211 showed 14% and 15% of SF, while NF2132 and NF235 indicated 19% and 18%, respectively. After 6 Gy, the lowest SF level was observed in NF202, resulting in 6% in contrast to NF211, which presented the highest SF value of 19% (*p* = 0.010). The rest of the NFs showed comparable results without indicating a statistical difference. What is more interesting is that the SF curves in NFs from different subtypes are closer to each other than in CAFs, which could suggest the effect of malignant cells. The statistical analysis of the coefficient of determination—R^2^ was performed—the higher it is, the better the regression line fits the data. The R^2^ for CAFs’ data were 0.77, whereas, for NFs, the parameter indicated 0.83. The statistical data sheet of obtained *p*-values is included in the Supplement Materials.

### 2.3. The DNA Double-Strand Breaks and p53 Expression after Proton IRR

**Figure 5:**
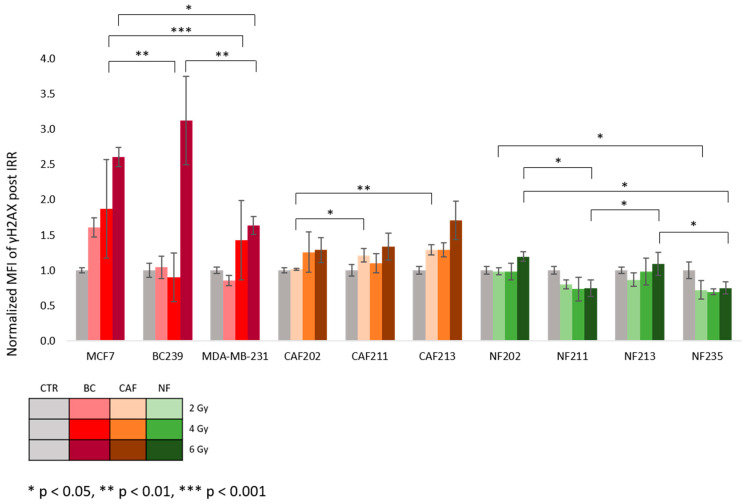
The mean fluorescence intensity (MFI) of γH2AX 1 h post proton IRR. After IRR, the γH2AX was labeled to indicate DNA (DSB). Cells were irradiated using doses of 2, 4, and 6 Gy. The results were compared between different cell lines, and each dose was analyzed. Various cell lines were: BC (red), CAF (orange), and NF (green). The *p*-value was adjusted to * *p* < 0.05, ** *p* < 0.01, and *** *p* < 0.001. The values presented in the graph are the mean of the obtained results and the deviation is the standard deviation. The experiment was performed in triplicate.

The parameters determining a radiobiological response include analysis of γH2AX, a commonly used biomarker for DNA double-strand breaks (DSB). Due to the DDR and quick repair of DSBs, all cells were fixed and permeabilized about 1 h post proton IRR to have the best insight into the primary damages. The obtained values were normalized to a control group. The results were analyzed using their origin type: BC, CAF, and NF (Figure 5). Within each category, the lines were compared. In most cell lines, a fluorescence intensity signal increased with the radiation dose. After a 2 Gy dose, there was no indication of differences between the BC cell lines. After a 4 Gy, we observed that the MCF7 cell line indicated a higher relative γH2AX level than the MDA-MB-231 and BC239 cell lines (*p* = 0.001, *p* = 0.004). The highest expression value was observed in the primary BC cell line BC239 after an IRR of 6 Gy. This γH2AX level was significantly higher than the commercially available TNBC line MDA-MB-231 *p* = 0.007. Moreover, considering the differences post 6 Gy, MCF-7 indicated an increased γH2AX level compared to the TNBC cell line (*p* = 0.046).

Meanwhile, CAF cell lines showed only weak dose responses with statistically significant differences after 2 Gy of IRR. CAF202 presented a significantly lower γH2AX level than CAF211 (*p* = 0.031) and CAF213 (*p* = 0.006). The higher doses of IRR did not generate varieties between CAFs, though a tendency to increase γH2AX level with a rising dose was observed.

Contrary to BC and CAF cells, NF cells showed no clear dose response. A decreasing trend for γH2AX level signal intensity with parallel increasing IRR doses. At the 2 Gy variant, the lower value was observed in NF235 vs. NF202 (*p* = 0.032), but when compared to NF211 and NF213, the differences were not significant. The 4 Gy of IRR did not demonstrate any diversity between irradiated NFs from different donors. The same signal intensity levels were observed in NF211 and NF235 after 6 Gy exposure. A similar observation was noted between NF202 and NF213. Both NFs, NF211 and NF235, were significantly lower compared to the NF202 and NF213 (*p* = 0.011, *p* = 0.034, respectively). The table containing all the data regarding statistical comparisons and obtained *p*-values is included in the Supplement Materials.

**Figure 6:**
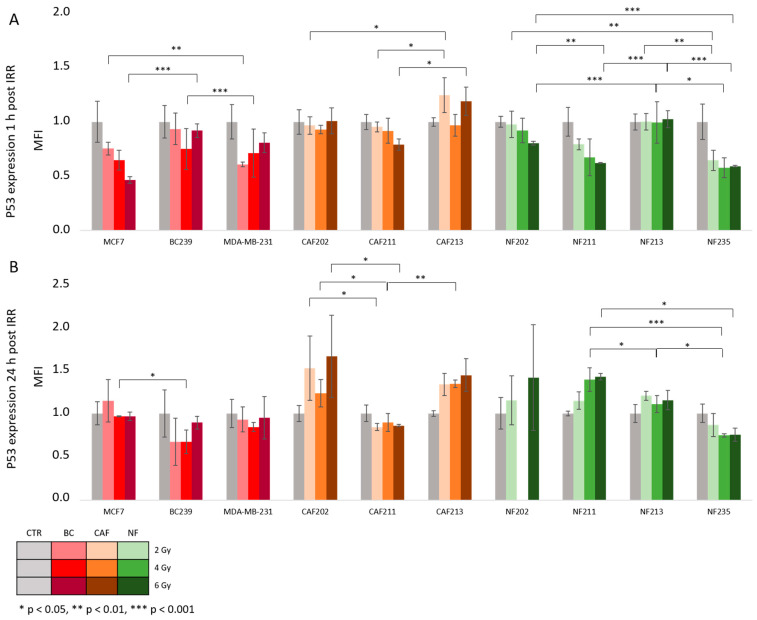
p53 expression 1 h (**A**) and 24 h (**B**) post IRR. All cells were irradiated with a proton beam using 2, 4, and 6 Gy doses. The results were compared between different cell lines analyzing each dose. A p53 value for NF202, 24 h post-IRR with a dose of 4 Gy, was missing because of the cell lack. Various cell lines were irradiated with a proton beam: BC (red), CAF (orange), and NF (green). The *p*-value was adjusted to * *p* < 0.05, ** *p* < 0.01, and *** *p* < 0.001. The values presented in the graph are the mean of the obtained results and the deviation is the standard deviation. The experiment was performed in triplicate.

The activation of the p53 protein is another radiobiological response indicator alongside γH2AX. After proton IRR, the p53 marker was analyzed at 2 time points (TP) at 1 h (Figure 6A) and 24 h (Figure 6B). The results were compared in the same way as it was performed for the γH2AX level. Interestingly, at 1 h TP, the p53 level indicated a decreasing trend with a higher dose of IRR. At 2 Gy a significant drop in the p53 level was noted between BC239 and MDA-MB-231 (*p* = 0.009). Between MCF-7 and BC239, these changes were not notable. The dose of 4 Gy did not induce any varieties between BC cells. The lowest p53 level was obtained by the MCF7 line at 6 Gy dose with a parallel lack of differences between BC239 and MDA-MB-231. The p53 signal level intensity was comparable with that of a control group after 24 h of exposure to proton IRR. Among tested doses, the only difference occurred at the 4 Gy dose, where MCF7 cells showed a higher p53 value than BC239 cells (*p* = 0.012).

The obtained data suggest that CAF cell lines indicated a similar response within the specific BC subtype from which they were derived. Thus, a comparable effect was observed between CAFs isolated from the tissue of luminal A tumors. CAF213 originating from TNBC indicated higher p53 signal intensity levels than CAF202 and CAF211 after 1 h post-IRR (*p* = 0.043, *p* = 0.036). Again, there was no difference between cell lines at the 4 Gy variant. Considering p53 levels post 6 Gy, a significant decrease in signal intensity was observed in CAF211 compared to CAF213 (*p* = 0.013). However, the changes in both of them were not significant in allocation to CAF202. After 24 h post-exposure, CAF202 and CAF213 showed some increasing trend in a dose-dependent manner, while in CAF211 there was a lack of changes in p53 signal levels intensity. Among CAFs derived from luminal A patients, at 2 Gy, a higher signal intensity was observed in CAF202 compared to CAF211 (*p* = 0.025). Between them and CAF213, there was a lack of significant changes. Similarly to 2 Gy, the differences between CAFs from the luminal subtype at 4 Gy were achieved (*p* = 0.032). On the other hand, at 6 Gy some apparent differences in signal intensity of p53 were noted in CAF202 (*p* = 0.036) and CAF213 (*p* = 0.007) compared with CAF211.

The overview of NF cell lines’ responses to the proton beam and their p53 signal intensity levels has shown the most significant differences. Besides NF213, all of NF has shown some decreasing trends in p53 signal intensity in a dose-dependent manner. Considering effects after 2 Gy of IRR among studied NFs, we observed that NF235, isolated from a patient diagnosed with TNBC, gained the lowest p53 level at 1 h post-IRR, which was significantly different compared to the NF202 and NF213 lines (*p* = 0.008, *p* = 0.005). Between NF235 and NF211, there was a lack of significant changes in its levels. At 4 Gy one hour post-IRR only significant differences were observed between NF213 and NF235 (*p* = 0.028). After 1 h at 6 Gy the highest level of p53 intensity was noted in NF213 (NF213 vs. NF202 *p* = 0.001; NF213 vs. NF211 *p* = 0.0001; NF213 vs. NF235 *p* = 0.0001). Moreover, the differences between NF211 and NF202 after 1 h were shown, which marked an increased level (*p* = 0.002, *p* = 0.001). After 24 h, the trends in NF202 and NF211 have changed into increasing ones with the dose-dependent matter. The NF213 and NF235 trends did not change. The differences after 24 h post-exposure to 2 Gy did not cause any difference among NF lines. Post-4 Gy, we noticed a reversed situation. It should be added that the number of compared NF lines was smaller, due to missed NF202 during experiments. The highest signal intensity of p53 was observed in the NF211 (NF211 vs. NF213 *p* = 0.029; NF211 vs. NF235 *p* = 0.001). In addition, in NF213, the p53 level signal intensity was higher than in NF235 (*p* = 0.010). 24 h post-exposure to 6 Gy, there was only one significant difference in its level between NF211 and NF235 (*p* = 0.046). The NF202 and NF213 did not show differences compared to NF211 or NF235. The statistical data sheet of obtained *p*-values is included in the Supplement Materials.

## 3. Discussion

This study evaluated the radiobiological response of established and primary BC cell lines, CAF and NF lines to proton IRR. We aimed to assess the obtained outcomes while considering two main aspects. Firstly, we wanted to compare the radiobiological responses by examining two molecular subtypes of BC: luminal A and TNBC. The former is generally regarded as a less aggressive BC form, contrasting with the latter, known for its high resistance to various therapies. Additionally, we intended to explore potential differences in the response to proton beams among non-cancerous lines isolated from patients diagnosed with a specific BC subtype. The second aspect involves comparing responses among lines of a particular cell type to determine whether they exhibit individualized responses, indicating a strong correlation with patient-specific factors, or whether it is a universal response despite cell type.

The initial phase involved assessing the biological model, which comprised two established tumor lines representing different molecular subtypes, one primary BC line, three CAF lines, and four NF lines, chosen to observe the response post-radiation. Through prior experience and optimization of the isolation process [15,16], we successfully verified and characterized the lines isolated from patients. Consistent with the previous studies [12,23], the isolated CAFs exhibited no subtype-specific differences; all lines displayed similar expression patterns of fibroblast markers. This trend was also evident in the NF line, although NF213 displayed notable results, suggesting unique characteristics possibly indicative of a specific patient, as its paired CAF line exhibited distinct marker levels compared to the others. An intriguing observation arose during the characterization of an isolated BC line. While histopathological analysis post-tumor resection indicated the luminal A molecular subtype, Western blot results revealed the TNBC subtype. It might be considered that the changes in tumor conditions during cell culture led to the loss of ER and PR receptors. This could be related to the possible loss of its expression due to the lack of presence of exogenous estradiol in the culture medium [24]. As the cells were irradiated at the same culture stage as the receptor analysis, we will categorize these lines as TNBC subtypes based on the obtained characteristics.

Based on SF analysis, it is noticeable that established BC lines of different molecular subtypes (luminal A and TNBC) displayed no significant variations in their response to proton IRR. This could imply that they exhibit similar reactions, which could be advantageous for the universal efficacy of proton therapy. The cancerous patient-derived cell line presented significantly lower SF than its commercially available counterparts. These differences emphasize the necessity for implementing a higher number of new biological models based on primary cell lines. To reassess this outcome, a more significant number of tumor lines would be required for IRR, but we widely recognized that isolating primary breast tumor lines poses a considerable challenge [16]. Regarding CAF lines isolated from the TME, the line obtained from a TNBC subtype tumor showcased increased resistance to ionizing radiation, while CAF202 and CAF211 exhibited comparable radiobiological responses. This might indicate that molecular factors associated with the BC molecular subtype from which the lines were derived could influence the response while not discounting patient-specific factors. Fibroblasts isolated from patients’ skin responded similarly across all doses of proton radiation. However, individual variances became apparent after radiation at a dose of 2 Gy, a level sufficient to detect disparities in response. As we previously discussed [3], numerous factors in both nearby and distant environments can impact a patient’s skin outcome. The dosage gradient present at the distant edge of the Bragg peak might heighten the risk of notable adverse effects. Patients may experience early or delayed effects that diminish their quality of life if radiation directly affects sensitive organs at risk near the treated tumor or triggers unintended responses. Bush et al. [25] studied the effects, including skin reactions, after proton treatment on 100 patients with invasive non-lobular carcinoma with a maximum tumor size of 3 cm. They administered a postoperative proton beam dosage of 40 Gy in 10 fractions once daily for two weeks. Patients were followed for 5 years after therapy to detect skin toxicity and tumor recurrence. Proton therapy resulted in 97% ipsilateral breast recurrence-free survival and 95% overall survival with minimal toxicity.

Cammarata et al. [26] aimed to examine the molecular reactions in response to proton RT and its effectiveness in a xenograft model of TNBC using MDA-MB-231 cells. Different doses of 2, 6, and 9 Gy were administered to TNBC xenograft models. Gene expression profiling (GEP) analyses and immunohistochemical assays were conducted to identify specific pathways and key molecules involved in the cellular response to IRR. The GEP analysis provided comprehensive insights into the molecular response to proton RT, revealing significant immune response modulation, regulation of cell cycle, and stem cell processes. It was observed that only the 9 Gy dose induced a shift towards pro-death signaling, which suggests a potential for dose escalation achievable with proton beams. Another group tested the crosstalk between CAFs and BC cells, considering radioresistance effects [27]. CAFs were observed to produce hepatocyte growth factor (HGF), stimulating the c-Met signaling pathway, thereby triggering epithelial-to-mesenchymal transition, proliferation, and resistance to radiation in BC cells. Furthermore, BC cells exposed to radiation released tumor necrosis factor α (TNFα), prompting CAF proliferation and HGF secretion. Notably, heightened expression of HGF and c-Met correlated with poorer recurrence-free survival among breast cancer patients who underwent radiation therapy. Targeting the HGF/c-Met signaling pathway emerges as a promising strategy for enhancing the radiosensitivity of breast cancer cells.

Protons interact with tissue fundamentally differently than photons, which lack both mass and charge. As a result, the induction of DNA damage and the mechanisms involved in DNA repair differ between proton and traditional RT [28]. One crucial aspect of describing the radiobiological response involves analyzing DNA DSBs indicated by γH2AX levels. Our study focused on the early response and DSB formation, in which maximum foci concentration per nuclei is presented between 30 min and 1 h post-IRR. We have chosen this time point due to the kinetic of γH2AX, where after 24 h even the residual damages could be detected, but not with decent differences [29,30,31]. A noticeable pattern of escalating damage levels in both BC and CAF lines with higher radiation doses exists. The BC239 line exhibited the highest level of damage after a 6 Gy dose, significantly predominating levels observed in established lines. This outcome is associated with a lower survival fraction, implying that more lesions diminished clonogenic capacity. Among CAF lines, variations in response emerged after a 2 Gy radiation dose; however, the levels of DNA DSBs were not markedly higher than those in the control group, suggesting that the reduction in proliferation might be attributable to factors other than DNA DSBs. There is a trend toward decreasing or consistent γH2AX levels among NF lines. In contrast to the survival fraction results, statistical disparities in γH2AX levels were noted at a radiation dose of 6 Gy. The scientists [32] presented that proton beam IRR results in a greater occurrence of both single and double-strand DNA breaks, reduced levels of H2AX phosphorylation, elevated Chk2 phosphorylation, and decreased cell cycle recovery from G2 arrest compared to photon radiation. This leads to the activation of caspase-3, cleavage of PARP, and, ultimately, apoptosis of cells. Additionally, proton IRR generates a substantial amount of reactive oxygen species (ROS), crucial in inducing DNA damage, redistributing the cell cycle, triggering apoptosis, and exerting cytotoxic effects.

Another molecular aspect considered in the analysis was the expression level of the p53 protein. What is worth mentioning is that the p53 response to IRR is not constant and could create a pulsating response despite the formed DSB [33]. Importantly, the response to irradiation is determined by the genetic background of the cancer. In this study, we have used MDA-MB-231 (mutant) and MCF-7 (wild type), whose responses are similar to the other research groups, meaning the TNBC has a higher tolerance to IRR than the luminal A type. Our cell line, BC239, despite the unknown p53 genetic status and some p53 protein expression, is radiosensitive, such as the histological type from which it was collected [34,35,36]. Moreover, the regulation of the p53 expression and its dynamic is also limited to the origin of the tissues and dictates the radiosensitivity [37,38]. Since we compare cancerous and normal cell lines with different cell cycle progression, we assessed this parameter at two time points: 1 and 24 h post-exposure to the proton beam. One hour after IRR, a clear trend in declining p53 levels with increasing radiation dose was evident. Notably, the CAF213 line and the NF213 line exhibited significantly different reactions compared to other lines of their respective types. Specifically, only CAF213 displayed elevated p53 expression values after IRR, whereas NF213 cells reached similar p53 levels as the control group. Once again, these anomalies point to a patient-specific radiobiological response, underscoring the importance of individualized therapy approaches that consider patient-specific recommendations. These particular factors likely stem from individually characteristic molecular factors.

Considering the results obtained 24 h post-IRR, a reverse trend in p53 levels was observed. In most instances, as the radiation dose increased, p53 levels rose. After radiation exposure, the DNA damage response triggers an elevation in p53 protein levels within cells by stimulating protein translation [39] and suppressing protein degradation [40]. The transcription factor p53 determines the radiation response; nevertheless, the abundance of p53 protein does not consistently align with the degree of radiosensitivity among tissues. These findings indicate that tissues sensitive to radiation exhibit prolonged p53 signaling following exposure, whereas less susceptible tissues display transient activation of p53 [37]. This was especially pronounced in the CAF202 and CAF213 lines, which exhibited higher values and were statistically distinct from CAF211, which showed lower values. Regarding the analysis of NF radiation results, it is worth noting that the p53 level result for a 4 Gy dose in the NF202 line is missing. During the analysis, a shortage of cells in the biological material was observed, and it was decided that the absence of a single result did not warrant excluding all results for this line. Repetition of the experiment was not feasible due to logistical constraints. Distinctive outcomes were noted in NF235, which was the only line to exhibit, as observed after 1 h, a decline in p53 levels with increasing dose. Other lines reacted similarly, once more suggesting that the model developed for normal lines is suitable for analyzing the effects of proton radiation. Comparable results were obtained by Oeck et al. [41]. Using prostate cancer and fibroblast cells, they measured the initial DNA damage, primarily DNA DSBs, and examined the repair kinetics by detecting γH2A.X or 53BP1 using immunofluorescence. While the initial counts of γH2A.X and 53BP1 foci were comparable across different IRR scenarios, it was noteworthy that the peak foci level was achieved at 60 min post-IRR with Bragg-peak protons, contrasting with 30 min for plateau protons and photons. Intriguingly, Bragg-peak protons induced larger and irregularly shaped γH2A.X and 53BP1 foci, with a delayed resolution of these foci.

Identifying molecular mechanisms between cells creating TME and BC is crucial, considering RT effects. It includes alterations in the stiffness of the tumor matrix and surrounding tissues. These changes have been associated with the development of radioresistance in BC cells and facilitate tumor recurrence and metastasis, thereby leading to unfavorable patient outcomes [42]. Scientists [43] explored the mechanism underlying tumor growth post-IRR and proposed a potential therapeutic approach. Fibroblasts were isolated from fresh tissue samples of nasopharyngeal carcinoma (NPC) and normal nasopharyngeal mucosa. They observed that radiation-resistant NPC tissues consistently preserved a higher population of CAFs than radiosensitive NPC tissues. Further investigation uncovered that CAFs enhanced the development of radioresistance and enhanced the survival of NPC cells following IRR through the IL-8/NF-κB pathway, which mitigated radiation-induced DNA damage.

A growing number of studies have suggested that RT should be based on patient-specific factors [44]. Even though data represent a clear correlation between the molecular subtype of BC and RT outcome, researchers highlight discrepancies in the analyzed parameters such as risk factors and side effects. This indicates the limitations in obtaining precise confirmation of the molecular subtypes’ impact on this therapy. The concept of personalized RT in BC can be divided into biology- and genomic-driven. The biology-associated personalization might be related to assessments of the impact of tumor molecular subtypes on the RT outcome and the possibility of early and late biological response of healthy tissues. The genomic-driven approach should be based on broad profiling that enables establishing well-defined genomic tests, composed of several genetic classifiers that enable risk stratification and radiosensitivity [44]. The RT outcome prediction could be established using information from large databases [45]. It might illustrate the correlation of an anatomic disease expansion with molecular BC subtypes. Targeting analyses linking genetic predispositions with response to radiotherapy could help qualify patients for a dedicated treatment modality.

Our study showed that a higher number of isolated cell lines would allow for a more accurate and reliable assessment of the correlation between molecular factors and radiobiological response. In the future, the analysis of quantitative and qualitative parameters after exposure to IRR might be broadened. Moreover, implementing the increased number of measurement time points could be considered due to DNA damage occurring differently over time. In addition, with the development of tissue engineering or lab-on-chip technology, more advanced and complex biological models can be used to test multiple patient tissues simultaneously. This would support the determination of the impact of tumor heterogeneity or better reproduction of tumor characteristics and response.

## 4. Methods

### 4.1. IRR of Tested Cells

**Figure 7:**
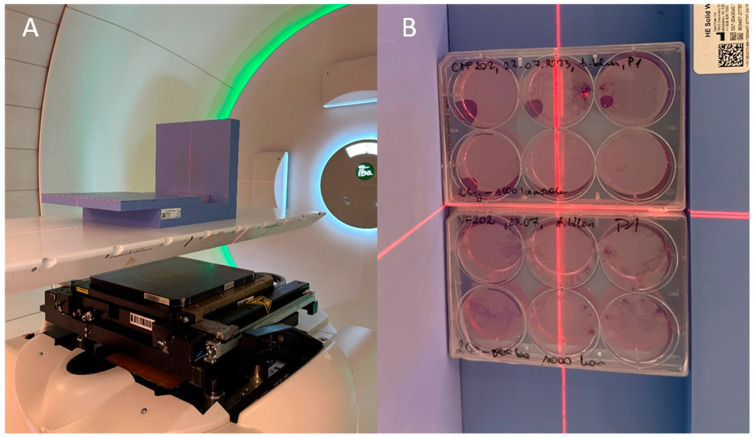
An example of an experiment that was set-up for cell IRR using a proton beam. This Solid Water^®^ High Equivalency phantom model enables IRR of cells cultured in 12- and 6-well plates. (**A**): Side view of positioned measurement set-up, (**B**): Top view of positioned measurement set-up.

Cells were placed in a designed solid water phantom made of Solid Water^®^ High Equivalency (Gammex Inc., Middleton, WI, USA), arranged in a phantom that allows all multi-well plates with cells to be irradiated in the same spacial configuration (Figure 7).

**Figure 8:**
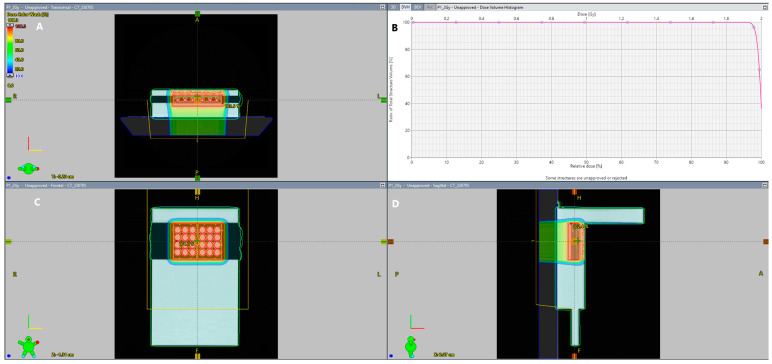
A representative scheme from planning 2 Gy irradiation of a 12-well plate using a modulated scanning technique. (**A**): transversal view, (**B**): dose-volume histogram, (**C**): coronal view, (**D**): sagittal view.

Treatment plans were prepared for two types of plates, 6-well and 12-well, assigned to clinical target volume (CTV). The CTV includes the cell holders with no margins. A radiation target volume (RTV) was created from the CTV with a symmetric set-up error of 0.3 cm and 3.5% uncertainty of the Hounsfield units (HU). A pencil scanning proton beam delivered doses of 2, 4, and 6 Gy to the cells with the IBA Proteus PLUS System (IBA Proton Therapy, Brussels, Belgium). The plans were prepared using a modulated scanning technique and consisted of one field with a range shifter of 3.5 cm water equivalent thickness (WET) inserted and an air gap of 13 cm for a 180 degree gantry angle (Figure 8). Optimization and calculations were performed in the Eclipse Treatment Planning System, v. 15.6 (Varian Medical Systems, Palo Alto, CA, USA), using a proton convolution superposition (PCS) dose algorithm and nonlinear universal proton optimizer (NUPO). The plan aimed to cover 98% of the CTV with 99% of the dose.

According to the clinical approach [46], dosimetry pre-verification was performed before IRR to determine the delivery accuracy of plans, using ionization chamber detector arrays MatriXX PT (IBA Dosimetry, Schwarzenbruck, Germany). Measurements were evaluated using the gamma method (gamma passing rate ≥ 95%, 3% dose difference, 2 mm distance to agreement, 10% dose cut-off, local approach).

### 4.2. Patients’ Material and Isolation

Patient inclusion criteria, the process of the biopsy materials collection, and cell culture isolation were determined in our previous article [16]. Shortly, patients diagnosed with invasive breast cancer (with a diameter of 15 mm or more) who were eligible for surgery were enrolled in the study. The study received ethical approval (number 283/21; 619/20) from the Bioethics Committee of Poznan University of Medical Sciences. CAF cell lines and BC239 cell line isolation were performed using ‘method 5’ presented by Janik et al. [47] and our further optimization [16]. Meanwhile, NF cell lines were isolated from breast skin according to the ‘isolation of normal primary fibroblasts’.

For this study, we used the primary NF cell lines, isolated from the same patient from whom the primary CAF cells were derived (patients numbered 202, 211, and 213), except for NF235. Patients were enrolled based on informed consent. Patients’ characteristics and molecular subtypes of tumors are included in Table 1.

### 4.3. Cell Culture

The following established BC cell lines representing molecular subtypes were used in this work: MDA-MB-231 (TNBC) and MCF7 (luminal A). MDA-MB-231 cells were cultured in DMEM (Biowest, Nauille, France) supplemented with 10% FBS (Biowest, France) and 1% penicillin/streptomycin (P/S) (Merck Millipore Corporation, Darmstadt, Germany). The MCF7 cell culture was provided using DMEM (Biowest, France) enriched with 10% FBS (Biowest, France), 1% P/S (Merck Millipore Corporation, Darmstadt, Germany), and 0.01 mg/mL insulin (Gensulin R, Bioton, Macierzysz, Poland). Isolated BC cell line BC239 was cultured with the same media as MCF7 cells. The culture of primary CAF and NF cell lines was performed with the same supplementation and media as the MDA-MB-231 cell line. The pictures were taken using an inverted microscope Axio Vert.A1 (Carl Zeiss, Oberkochen, Germany) at 100× magnification.

### 4.4. Primary Cell Line Phenotyping

Harvested CAF, NF, and BC239 cells were suspended in phosphate-buffered saline (PBS) and washed twice. Then, cells were incubated with PBS containing 2% FBS and 5 μL of the antibodies for extracellular staining: CD24 (catalog number: #B23133), CD31 (catalog number: #B13035), CD44 (catalog number: #B37789), CD45 (catalog number: #IM2473U), CD90 (catalog number: #IM1839), CD326 (#1A-582-T100), CD140a (catalog number: #1P-589-T100), CD140b (catalog number: #1P-590-T100), and CD29 (catalog number: #1F-219-T100) (EXBIO, Praha, Czech Republic) for 30 min at 4 °C. Following that, PBSwas used to wash the cells once. The intracellular staining was performed using The Fixation/Permeabilization Kit (BD Biosciences, Franklin Lakes, NJ, USA) according to manufacturer instructions. The BC239 cells were stained with Vimentin (VIM) (catalog number: #1A-369-C100) and E-Cadherin (E-CAD) (catalog number: #1F-588-T100) (EXBIO, Czech Republic) antibodies. After that, cells were washed with PBS and incubated for 30 min at 4 °C with the antibody. The Cytoflex Beckmann Coulter cytometer (Beckman Coulter Life Sciences, Brea, CA, USA) was used to read out the signal. FlowJo v10 (FlowJo LLC, Ashland, OR, USA) was used to analyze the obtained results.

### 4.5. Clonogenic Assay

Before IRR (used doses: 2, 4, and 6 Gy), cells were plated into 6-well plates: 500 cells per well for the control group (0 Gy) and an increasing number corresponding to the increase in the doses: 1000, 1500, and 2000 cells per well, respectively. After 24 h, cells were irradiated in the proton facility, transported, and kept in the incubator for 7–8 days, depending on the cell line. The assay was stopped when a colony formed from at least 50 cells was seen in the control group. Then, the cells were washed with PBS and treated with ethanol for fixation. To visualize the cells, they were incubated for 20 min in 2 mL of Coomassie Blue solution (Merck Millipore Corporation, Darmstadt, Germany). The plates were dried and cleaned in warm water to decrease the background. The images of the plates were documented using the ChemiDoc Touch Bio-Rad system (Hercules, CA, USA). Colony counting was carried out automatically using the Gene Tools Syngene program. After counting the colonies, plating efficiency (PE) was calculated, expressing the ratio of counted colonies to seeded cells. The surviving fraction (SF) was then calculated as the ratio of PE of irradiated cells and PE of control cells.

### 4.6. Flow Cytometry Analysis after IRR

For flow cytometry analysis, the cells were seeded on 12-well plates. After 24 h, cells were irradiated under the same conditions as those irradiated for clonogenic assays.

Following 1 h (γH2AX and p53) and 24 h (p53) after IRR, 200,000 cell samples were fixed and permeabilized using the Fixation/Permeabilization Kit (BD Biosciences, NJ, USA) according to the manufacturer protocol. The prepared cells were treated with anti-γH2AX antibody (Becton Dickinson, Franklin Lakes, NJ, USA) or anti-p53 antibody (catalog number: #1F-114-C100) (EXBIO, Czech Republic). The Cytoflex Beckman Coulter cytometer (Beckman Coulter Life Sciences, ID, USA) was used to measure the signal of stained cells. FlowJo v10 (FlowJo LLC, USA) was used to analyze the obtained results.

### 4.7. Quantitative PCR

Using Direct-zol RNA MiniPrep (Zymo Research, Irvine, CA, USA), total RNA was extracted from 500,000 cells previously suspended in TRI reagent (Sigma-Aldrich, St. Louis, MO, USA). 1 μg of total RNA was reverse-transcribed using the iScript kit (Bio-Rad, Hercules, CA, USA). The cDNA was diluted in deionized molecular grade H_2_O (Sigma-Aldrich, St. Louis, MO, USA) in a total amount of 200 μL. The gene expression was evaluated by RT-qPCR using probes. The human GAPDH gene was used as a reference gene to calculate relative expression. All primers used in the study are shown in Table 2. The qPCR reaction and Cq values were estimated using the CFX96 Touch Real-Time Detection System (Bio-Rad, Hercules, CA, USA) in the standard protocol as previously described [16].

### 4.8. Western Blot

The receptor status and p53 of the tested cell lines (MDA-MB-231, SKBR3, T47D, MCF-7, BC239 (malignant), and MCF12A (normal)) were confirmed using the Western blot technique as previously described [48]. In brief, the protein was isolated from 1 × 10^6^ malignant or normal breast cell lines using RIPA buffer (Sigma-Aldrich, MO, USA), and its concentration was determined by BCA assay (Thermo Fisher Scientific Inc., Waltham, MA, USA). The mixture of Laemmli sample buffer and 10 µg of protein was denatured at 99 °C for 10 min, loaded onto the precast polyacrylamide gel (4–15% gradient), and then transferred to a membrane (PVDF, polyvinylidene difluoride) (all provided from Bio-Rad Laboratories Ltd., CA, USA). After that, the membrane was blocked for 2 h at room temperature with TBST with 5% non-fat milk (Sigma-Aldrich, MO, USA) and incubated overnight at 4 °C with primary antibodies (antibodies dilution: anti-PRA—1:500, Novus Biological, LLC, CO, USA; GAPDH—1:500 Santa Cruz, CA, USA; anti-ERα—1:1000, Cell Signaling Technology, Danvers, MA, USA; anti-HER2—1:1000 Novus Biological, LLC, CO, USA; anti-p53—1:1000, Cell Signaling Technology, Leiden, The Netherlands). On the next day, the membrane was washed and then incubated with secondary antibodies conjugated with horseradish peroxidase (antibodies dilution: anti-mouse IgG or anti-rabbit IgG—1:5000; Cell Signaling Technology, Leiden, The Netherlands). The labeled proteins were detected using Clarity Western ECL, and signal readout was performed using the ChemiDoc Touch Imaging System (Bio-Rad Laboratories Ltd., Hercules, CA, USA). The signal intensity was determined using ImageLab ver. 6.1.0 (Bio-Rad Laboratories Ltd., Hercules, CA, USA).

### 4.9. Statistical Analysis

The statistical analysis was conducted using Microsoft Office Professional Plus 2019 and PQStat Software v.1.8.2. The observed data distribution’s normality was assessed using the Shapiro–Wilk test. One-way ANOVA was applied to determine the differences for a complex system (more than two groups), and Tukey’s post hoc test was performed to make multiple comparisons. The Welch’s *t*-test, also known as the unequal variance t-test, was used if Levene’s test revealed that the variances were unequal among groups. The data were significant when the *p*-value = 0.05 was employed. The following asterisks described the significant changes between groups: * *p* ≤ 0.05, ** *p* ≤ 0.01, and *** *p* ≤ 0.001.

## 5. Conclusions

This study underscores that tumor response is not solely dependent on eliminating breast cancer cells, but also enhances the role of tumor microenvironmental factors and skin reactions. Response of patient-derived cell lines after proton beam exposure indicated that each patient might demonstrate different RT outcomes because of various tumor microenvironment-related factors. We showed that cancer-associated fibroblasts are a new model reflecting the individual response to the proton RT, shedding new light on future IRR models. In contrast, analysis of skin response revealed a consistent outcome of normal fibroblasts, offering promise for establishing a comprehensive standard treatment approach. This study highlights the importance of each tissue-type interaction with proton beams in designing personalized RT protocols.

## 6. Ethics Approval and Consent to Participate

The study received ethical approval (number 283/21; 619/20) from the Bioethics Committee of Poznan University of Medical Sciences.

## Figures and Tables

**Table 1:** Patients’ data and pathology results of used BC biopsy samples.

Cell Line	Grade	Age	Side	ER	PR	HER2+	Ki67	BC Subtype
CAF202 NF202	NHG2	79	Right	+	+	−	10%	Luminal A
CAF211 NF211	NHG2	54	Left	+	+	−	10%	Luminal A
BC239	NHG1	72	Right	+	+	−	10%	Luminal A
CAF213 NF213	NHG3	89	Right	−	−	−	70%	TNBC
NF235	NHG3	71	Right	−	−	−	80%	TNBC

Abbreviations: CAF—cancer-associated fibroblasts, NF—normal fibroblasts, NHG—Nottingham histological grade, ER—estrogen receptor, PR—progesterone receptor, HER2—human epidermal growth factor receptor 2, Ki67—antigen kiel 67, BC—breast cancer, TNBC—triple negative breast cancer.

**Table 2:** Primers used in the study.

Gene	Probe	Forward	Reverse
GAPDH	n/a	TCCACTGGCGTCTTCACC	GGCAGAGATGATGACCCTTTT
CD24	68	AGCCTACTGCAAATCCAAACA	GAAGCTCTGAGAATTACTCTGCTG
NANOG	31	ATGCCTCACACGGAGACTGT	AAGTGGGTTGTTTGCCTTTG
COL1A2	18	CACTCCTGGCACTGATGGT	CATTCCCTGAAGACCTGGAG
SNAIL1	62	GCTGCAGGACTCTAATCCAGA	ATCTCCGGAGGTGGGATG
MMP2	67	ATGCCGCCTTTAACTGGAG	GGAAGCCAGGATCCATTTTC
VIM	24	GGGACCTCTACGAGGAGGAG	CTTTGTCGTTGGTTAGCTGGT
CD44	31	AGCCTACTGCAAATCCAAACA	GAAGCTCTGAGAATTACTCTGCTG
CDH1	77	AAGTTTTCCACCAAAGTCACG	TGCTTGGATTCCAGAAACG
TP53	71	CTTTCCACGACGGTGACA	TCCTCCATGGCAGTGACC

Abbreviations: n/a—not appicable.

## Data Availability

Data sharing is not applicable to this article as no datasets were generated or analyzed during the current study.

## Supplementary Materials

The following supporting information can be downloaded at: https://www.mdpi.com/article/10.3390/ijms251910494/s1.
