# Supplementary material for: Impact of Proton Irradiation Depending on Breast Cancer Subtype in Patient-Derived Cell Lines
Source: Int J Mol Sci. 2024 Sep 29;25(19):10494. doi: 10.3390/ijms251910494 (PMC11477436; doi:10.3390/ijms251910494)
Supplement: Supplementary file 1 [file ijms-25-10494-s001.zip › Figure S1. Phenotyping of isolated CAF and NF cell lines.pdf]

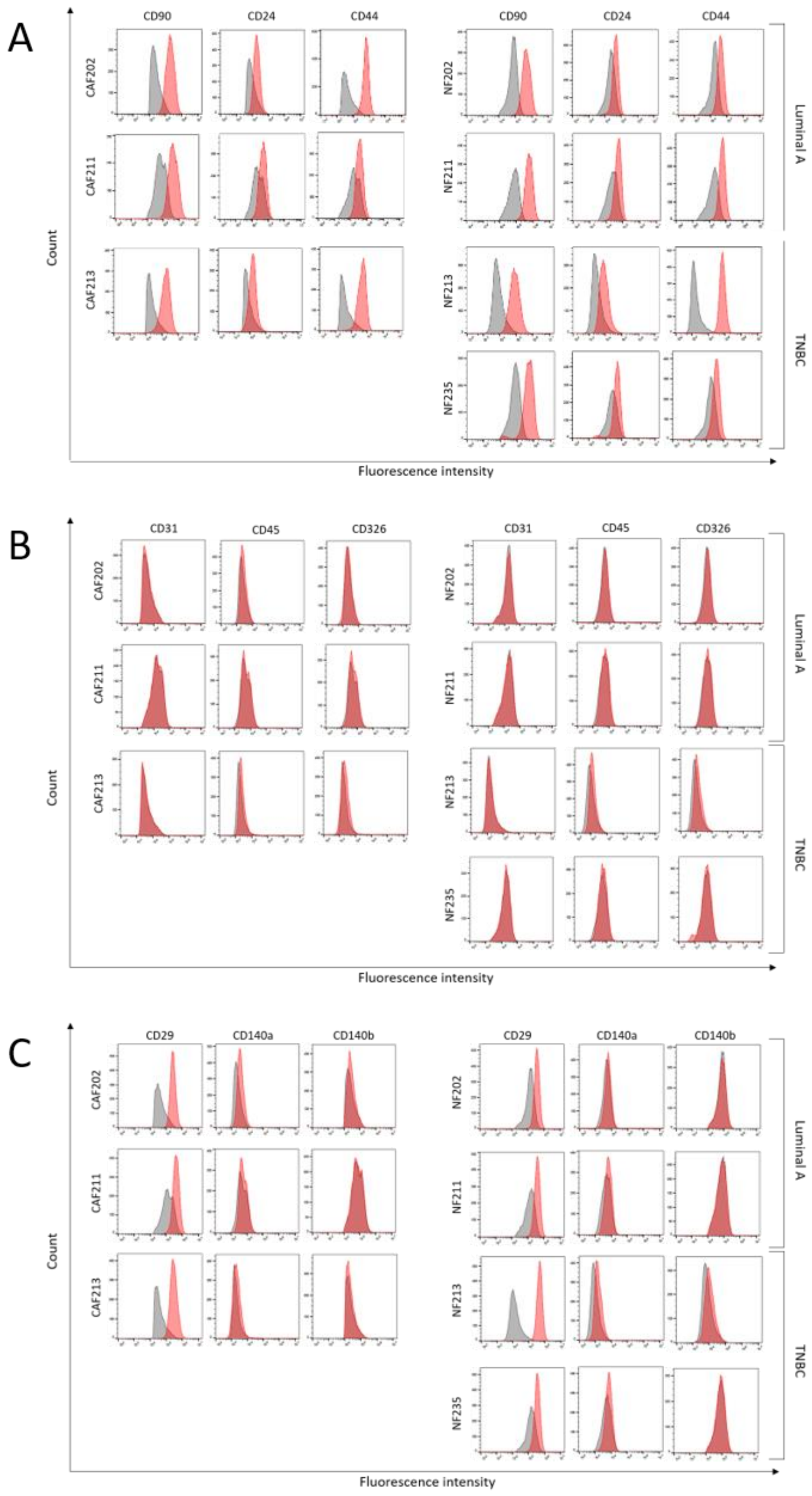

Figure S1. Phenotyping of isolated CAF and NF cell lines. A: A preliminary phenotyping of every cell line. To validate their fibroblast phenotype, CAFs and NFs obtained from patients were characterized using CD24, CD44, and CD90,. The examination of CD24 expression was conducted to assess the possibility of epithelial cell contamination. The CD90 antibody determined whether cells indicate the fibroblast origin. Depending on

patients' specific characteristics, the CD44 expression in cells might be observed. B: Analysis excluding other cell types using CD31, CD45, and CD326 biomarkers. It has been established that CD31 - negative cells are not endothelium, CD45 - negative cells are not leukocytes, and CD326 – negative cells were not epithelial. C: Characterization of cell molecular subtypes, including CAF-characteristic markers. CD140a (PDGFRα), and CD140b (PDGFRβ) are usually detected in all CAFs. Integrin beta-1, a cell surface receptor encoded by the ITGB1 gene in humans, is marked by CD29. These integrins combine to produce integrin complexes, which act as receptors for collagen. Grey histograms represent isotype control.
